# Novel metabolic and lipidomic biomarkers of sarcopenia

**DOI:** 10.1002/jcsm.13567

**Published:** 2024-08-21

**Authors:** Wei‐Hsiang Hsu, San‐Yuan Wang, Yen‐Ming Chao, Ke‐Vin Chang, Der‐Sheng Han, Yun‐Lian Lin

**Affiliations:** ^1^ Department of Chinese Pharmaceutical Sciences and Chinese Medicine Resources China Medical University Taichung Taiwan; ^2^ Institute of Biopharmaceutical Sciences National Yang‐Ming‐Chiao‐Tung University Taipei Taiwan; ^3^ Master Program in Clinical Genomics and Proteomics, College of Pharmacy Taipei Medical University Taipei Taiwan; ^4^ Department of Pharmaceutical Sciences, School of Pharmacy, College of Pharmacy Taipei Medical University Taipei Taiwan; ^5^ Department of Physical Medicine and Rehabilitation National Taiwan University Hospital, Bei‐Hu Branch Taipei Taiwan; ^6^ Department of Physical Medicine and Rehabilitation National Taiwan University College of Medicine Taipei Taiwan; ^7^ Health Science and Wellness Center National Taiwan University Taipei Taiwan; ^8^ Department of Pharmacy National Taiwan University Taipei Taiwan

**Keywords:** Biomarker, Lipidome, Metabolome, Nutrition, Resistance training, Sarcopenia

## Abstract

**Background:**

The pathophysiology of sarcopenia is complex and multifactorial and has not been fully elucidated. The impact of resistance training and nutritional support (RTNS) on metabolomics and lipodomics in older adults with sarcopenia remains uncertain. This study aimed to explore potential biomarkers of sarcopenia and clinical indicators of RTNS in older sarcopenic adults.

**Methods:**

Older individuals diagnosed with sarcopenia through routine health checkups at a community hospital were recruited for a 12‐week randomized controlled trial focusing on RTNS. Plasma metabolomic and lipidomic profiles of 45 patients with sarcopenia and 47 matched controls were analysed using ^1^H‐nuclear magnetic resonance (^1^H‐NMR) and liquid chromatography‐mass spectrometer (LC–MS).

**Results:**

At baseline, the patient and control groups had similar age, sex, and height distribution. The patient group had significantly lower weight, BMI, grip strength, gait speed, skeletal muscle index, lean mass of both the upper and lower limbs, and lower limb bone mass. There was a significant difference in 12 metabolites between the control and patient groups. They are isoleucine (patient/control fold change [FC] = 0.86 ± 0.04, *P* = 0.0005), carnitine (FC = 1.05 ± 0.01, *P* = 0.0110), 1‐methylhistamine/3‐methylhistamine (FC = 1.24 ± 0.14, *P* = 0.0039), creatinine (FC = 0.71 ± 0.04, *P* < 0.0001), carnosine (FC = 0.71 ± 0.04, *P* = 0.0007), ureidopropionic acid (FC = 0.61 ± 0.10, *P* = 0.0107), uric acid (FC = 0.88 ± 0.03, *P* = 0.0083), PC (18:2/20:0) (FC = 0.69 ± 0.03, *P* = 0.0010), PC (20:2/18:0) (FC = 0.70 ± 0.06, *P* = 0.0014), PC (18:1/20:1) (FC = 0.74 ± 0.05, *P* = 0.0015), PI 32:1 (FC = 4.72 ± 0.17, *P* = 0.0006), and PI 34:3 (FC = 1.88 ± 0.13, *P* = 0.0003). Among them, carnitine, 1‐methylhistamine/3‐methylhistamine, creatinine, ureidopropionic acid, uric acid, PI 32:1, and PI 34:3 were first identified. Notably, PI 32:1 had highest diagnostic accuracy (0.938) for sarcopenia. 1‐Methylhistamine/3‐methylhistamine, carnosine, PC (18:2/20:0), PI 32:1, and PI 34:3 levels were not different from the control group after RTNS. These metabolites are involved in amino acid metabolism, lipid metabolism, and the PI3K‐AKT/mTOR signalling pathway through the ingenuity pathway analysis.

**Conclusions:**

These findings provide information on metabolic changes, lipid perturbations, and the role of RTNS in patients with sarcopenia. They reveal new insights into its pathological mechanisms and potential therapies.

## Introduction

The prevalence of sarcopenia is from 13% to 24% in individuals aged larger than 65 years and has become a great economic burden in the aged society.^1^ Sarcopenia is characterized by low muscle mass and strength, and low physical performance.^2^ It is associated with poor clinical outcomes including falls, fractures, functional decline, frailty, and mortality.^2^, ^3^

The causes of age‐related sarcopenia are multifactorial involving myostatin, inflammatory cytokines, and mitochondria‐derived dysfunction; however, the underlying molecular mechanisms remain largely unclear.^4^ In particular, age‐induced mitochondrial dysfunction triggers the production of reactive oxygen species (ROS), disrupts mitochondrial dynamics, hinders mitophagy, and leads to mitochondria‐mediated apoptosis. The decline in muscle mass and function could be attributed to reduced physical activity, decreased energy intake, poor nutritional status, compromised immunity, and metabolic disturbances.^5^ The availability of preventive and therapeutic interventions for sarcopenia is currently limited,^3^ with lifestyle modifications, including protein supplementation and resistance training being the most successful approach.^6^ Resistance training helps increase muscle mass, strength, and endurance by subjecting muscle to repetitive load near the maximal muscle strength. The contraction of skeletal muscle with repetitive high load releases cytokines and small‐molecule metabolites. Shin et al. reported L‐alanine, gluconic acid, proline, and tryptophan may be potential blood biomarkers of severe sarcopenia through metabolomic analysis.^7^ Our previous study found that early exercise and nutritional intervention could improve lean mass, grip strength, and gait speed in patients with sarcopenia.^8^ However, the metabolomic profiles of older individuals with sarcopenia undergoing early exercise and nutritional intervention remain poorly understood.

Metabolomics is an approach used to comprehensively profile the presence of small molecules (<1500 Dalton) in biofluids, cells, and tissues, such as amino acids, lipids, carbohydrates, organic acids, nucleic acids, and vitamins, and to quantitatively measure the dynamic multi‐parametric metabolic responses of living systems to pathophysiological stimuli.^9^ This approach is instrumental in understanding the pathogenesis and diagnosis of diseases^10^ and evaluating complex disease mechanisms.^11^ Lipidomics, a newly established sub‐discipline of metabolomics, focuses on the study of cellular lipids on a large scale, and lipidome changes in living systems in response to internal and external perturbations, providing further insights into the complex pathophysiology of diseases.^12^ Using a metabolomics approach to identify potential markers of sarcopenia proves to be a reasonable and efficient strategy.^13^

This study is an expanded investigation on the plasma metabolomic and lipidomic profiles of the randomized controlled trial (NCT02779088) in older patients with sarcopenia.^8^ The aim of this study was to compare the untargeted plasma metabolome and lipidome of older adults with and without sarcopenia using ^1^H‐NMR, LC‐QTOF‐MS, and LC–MS/MS. Employing both NMR and MS ensure a comprehensive analysis of a wide range of metabolites. Furthermore, we attempted to investigate metabolic changes in patients with sarcopenia undergoing resistance training and nutritional support (RTNS). We identified the principal metabolites and metabolic pathways exhibiting significant differences between the sarcopenia and control groups. These findings hold potential to serve as clinical indicators of the efficacy of RTNS in managing sarcopenia.

## Materials and methods

### Participants, settings, and clinical evaluations

The study is the post‐hoc analysis of a randomized controlled trial, including individuals aged 65 years and older who participated in a geriatric annual health checkup in National Taiwan University Hospital, Bei‐Hu Branch. They were required to have normal cognitive function and to walk without assistance. Participants with atrial fibrillation, atrial flutter, ventricular bigeminy, cardiac pacemakers, malignancy, severe infection, coagulopathy, or known uncontrolled medical conditions were excluded from the study. Controls without sarcopenia were selected from the same group and matched for age and sex.^8^ The study protocol was approved by the Institutional Review Board of National Taiwan University Hospital (201601091RIND) and registered in ClinicalTrials.gov (NCT02779088) on 20 May 2016. All participants provided written informed consent before entering the study. All clinical investigations were conducted according to the principles of the Declaration of Helsinki.

### Diagnosis of sarcopenia

Sarcopenia was defined according to the Asian Working Group for Sarcopenia (AWGS) 2019 consensus criteria, which requires low muscle mass and either low muscle function or reduced physical performance.^14^ Low muscle mass was defined as a skeletal muscle mass index < 7.0 kg/m^2^ for men or <5.7 kg/m^2^ for women, while low muscle function was defined as handgrip strength less than 28 kg in men or 18 kg in women. Reduced physical performance was defined as gait speed lower than 1.0 m/s.^12^

### Grip strength, gait speed, and body composition

The measurement of grip strength, gait speed, and body composition was mentioned previously.^8^ The details were described in Data S1.

### Supervised resistance training and nutritional support

Following baseline evaluation, participants were randomly assigned to either the early or delayed intervention groups using simple randomization. This study was a parallel‐group, open‐label, randomized controlled trial consisting of two phases, each lasting 3 months, and with a 2‐week interval between them, which was reported previously.^8^ One phase involved supervised resistance training and nutritional support (RTNS), while the other involved home‐based exercises. In the early intervention group, RTNS were administered first, followed by a home‐based programme. In contrast, this sequence was reversed in the delayed‐intervention group. Participants were evaluated at baseline and immediately after each phase. The metabolomic and lipidomic profiles were measured before and after 12 weeks of RTNS intervention.

During the supervised RTNS periods, participants visited the Department of Physical Medicine and Rehabilitation, National Taiwan University Hospital Beihu Branch twice per week for 12 weeks. The RT involved pneumatic resistance training machines (Keiser Sports Health Equipment, Fresno, CA, USA) and the 1 repetition maximum (RM) of the leg press, leg extension, and leg curl was measured by an experienced physical therapist. The training programme started with a 10‐min warm‐up exercise, followed by three sets of 10 repetitions each, beginning at 40% of the 1 RM. Participants were asked to rest for 2 min between sets and then cool down for 10 min on a stationary bike. One RM was re‐evaluated weekly, and the training intensity was progressively increased up to a maximum of 80% of 1 RM, based on individual tolerance.

Nutritional support included two sticks of branched‐chain amino acids (BCAA‐Amino Vital Pro®, Ajinomoto) and two tablets of calcium and vitamin D3 supplements (Caltrate, Pfizer, USA) per day for 12 weeks. Each stick contained leucine, isoleucine, valine, glutamine, and other amino acids, while each tablet contained 800 IU of cholecalciferol and 600 mg of calcium.

### Blood collection

Each participant underwent peripheral blood collection of 10 ml from the antecubital vein upon recruitment. For sarcopenia patients, samples were collected both before and after the 12‐week RTNS programme, while for control cases, samples were collected on the day of the health check‐up and after at least 8 h of overnight fasting. Blood samples were collected in EDTA tubes on ice. The plasma was immediately separated by centrifugation at 1500 *g* for 20 min at 4°C. The samples were then stored in 500 μL aliquots at −80°C until analysis.

### Metabolomics and lipidomics profiling of the plasma samples

The ^1^H‐NMR metabolomics profiling was run at the NMR Core, BioTReC, Academia Sinica, on a Bruker Ascend 600 MHz NMR with a regular probe. The analysis was performed mainly as previously described using 200 μL of plasma in 300 μL D_2_O.^15^ Each spectrum was segmented into bins with a width of 0.005 ppm (2.5 Hz) between 0.2 and 10.0 ppm, using custom‐written ProMetab version 3.3 (Viant 2007) in MATLAB and removed the water peaks (from 4.7 to 5.0). The total spectral area of the remaining bins was normalized to unity to facilitate comparison between spectra. The area within each spectral bin was integrated to yield intensity‐based descriptors of the original spectrum. The binned data were subjected to the generalized log transformation, and the columns were mean‐centred before multivariate analysis.

LC–MS‐based metabolomics and lipidomics analysis were conducted at the Metabolomics Core Laboratory of the Center of Genomic and Precision Medicine, National Taiwan University. For metabolite extraction, 100 μL of plasma was mixed with 400 μL of methanol, homogenized at 1000 rpm for 2 min, and centrifuged at 15 000 rcf for 5 min at 4°C. The supernatant (380 μL) was dried, reconstituted with 200 μL of 50% methanol, centrifuged again, filtered using a 0.2 μm Minisart RC4 filter (Sartorius Stedim Biotech), and transferred to a glass insert for metabolomics profiling. For lipid extraction, 100 μL of plasma was mixed with 100 μL water and 1000 μL methanol/chloroform (1:2), shaken at 1000 rpm for 5 min using a Geno/Grinder 2010 (SPEX SamplePrep.), and centrifuged at 15 000 rcf for 5 min at 4°C. The lower organic layer (600 μL) was dried under nitrogen, reconstituted in 120 μL methanol, sonicated for 10 min, centrifuged again, filtered using a 0.2 μm Minisart RC4 filter (Sartorius Stedim Biotech), and transferred to a glass insert for lipidomics profiling.

Metabolomic profiling was performed using an Agilent 1290 UHPLC system coupled with an Agilent 6540 QTOF (Agilent Technologies). A 2 μL sample was injected into an Acquity HSS T3 column (2.1 × 100 mm, 1.8 μm, Waters) maintained at 40°C. The mobile phase consisted of solvent A (water/0.1% formic acid) and solvent B (acetonitrile/0.1% formic acid). The gradient elution programme was as follows: 0–1.5 min: 2% B; 1.5–9 min: linear gradient from 2% to 50% B; 9–14 min: linear gradient from 50% to 95% B; and 3 min maintenance in 95% B. The flow rate was 300 μL/min. A jet stream electrospray ionization source was used for ionization. The following parameters were used throughout the study: 325°C gas temperature, 8 L/min gas flow, 40 psi nebulizer, 325°C sheath gas temperature, 10 L/min sheath gas flow, 4000 V in positive mode and 3500 V in a negative mode for capillary voltage, and 120 V fragmentation voltage. The mass scan range and acquisition rate were m/z 50–1700 and 2 Hz, respectively.

The LC‐QTOF‐MS raw data were converted to mzXML format using ProteoWizard msConvert, and the detected abundant chromatographic peaks was normalized for further statistical analysis. The metabolite identification was performed by matching *m/z* value and retention time of each detected chromatographic peak to the National Taiwan University MetaCore In‐house Metabolomics Library, which consists of 383 metabolites.

Lipidomic profiling was performed using an Agilent 1290 UHPLC system coupled with a Bruker maXis impact QTOF (Bruker Daltonik). A 2 μL sample was injected into an Agilent ZORBAX Eclipse Plus C18 column (2.1 × 100 mm, 1.8 μm, Agilent Technologies) maintained at 55°C. The mobile phase was composed of solvent A (water/10 mM ammonium acetate/0.1% formic acid) and solvent B (methanol: isopropanol: water = 60: 40: 1/10 mM ammonium acetate/0.1% formic acid). The gradient elution programme was as follows: 0–2 min: linear gradient from 35 to 80% B; 2–7 min: linear gradient from 80 to 100% B; and 10 min maintenance in 100% B. The flow rate was 350 μL/min. For sample ionization, an electrospray ionization source was used. The following parameters were used throughout the study: 180°C dry gas temperature, 8 L min^−1^ dry gas flow, 2.0 bar nebulizer, 500 V end plate offset, 4500 V in positive mode for capillary voltage. The mass scan range and acquisition rate were m/z 150–1600 and 2 Hz, respectively.

The LC–MS/MS data were converted to mzXML format using ProteoWizard msConvert and the PITracer method^16^ was used to detect the chromatographic peak. The scaling‐based normalization was performed according to the total ion abundances from each LC–MS/MS dataset.^17^ The lipid identification was performed by matching m/z value to National Taiwan University MetaCore In‐house Lipdomics Library, which consists of sphingomyelins (SMs), lysophosphatidylcholine (LPC), ceramides (Cers), phosphatidylcholines (PCs), phosphatidylinositol (PI), phosphatidylethanolamine (PE), and cerebroside (CB).

### Statistics

For metabolomics and lipidomics, the normalized datasets were exported to SIMCA‐P + v12.0 (Umetrics, Umeå, Sweden) or MetaboAnalyst 5.0 (http://www.metaboanalyst.ca) for multivariate statistical analysis, principal component analysis (PCA), and orthogonal partial least squares discriminant analysis PLS‐DA (OPLS‐DA). These analyses aimed to identify the metabolites that contributed the most to distinguishing sarcopenia patients from controls. Using a variable importance in projection (VIP) cutoff value of 1, we determined whether the metabolites were potential sarcopenia‐relevant signatures. To increase the reliability of sarcopenia prediction, we calculated the receiver operating characteristic (ROC) curve based on a logistic regression model to determine the area under the ROC curve (AUC). Sensitivity, specificity and cut‐off value were obtained through ROC curve analysis. IBM SPSS 23.0 was used to analyse correlations between clinical parameters and targeted metabolites. Descriptive statistics are presented as mean ± standard error of the mean (SEM), median (range), or number (percentage). Student's *t*‐tests were used to compare groups, as appropriate. All calculated *P* values were two‐tailed. *P* < 0.05 was considered statistically significant. Differences were evaluated using the Student's *t*‐test, with *P* < 0.05 as the significance threshold. All *P* values are corrected as FDR‐adjusted *P* values using Benjamini–Hochberg procedure.

### Bioinformatics analyses

Ingenuity pathway analysis (IPA) software (Ingenuity Systems, Mountain View, CA, USA), MetaboAnalyst 5.0 (http://www.metaboanalyst.ca), and ConsensusPathDB (CPDB) (http://cpdb.molgen.mpg.de/) were employed to analyse biological pathways and functional annotations of metabolomics or proteomics data.

## Results

### Untargeted metabolomic and lipidomic profiling of plasma from sarcopenic patients

The study was conducted over a period of 6 months, from March to September, 2018. Of the 66 individuals diagnosed with sarcopenia during the annual geriatric examination, 58 agreed to participate and provided informed consent. One participant withdrew and did not attend follow‐up evaluation. Finally, 57 participants completed the course of supervised resistance training, and 57 age‐ and sex‐matched older individuals without sarcopenia were recruited as controls. Among them, 45 participants (male/female = 11/34) and 47 controls (male/female = 12/35) underwent full metabolomic and lipidomic analyses. At baseline, both groups had similar age, sex, and height. However, the intervention group had lower weight, BMI, grip strength, gait speed, skeletal muscle index, and lean mass of both the upper and lower limbs (Table 1).

**Table 1:** Demographics, physical performance and body composition of the control and patient group at baseline

	Control		Patient		*P* value
	*N* (%)	Mean ± SD	*N* (%)	Mean ± SD	
*N*	47		45		
Demographic					
Sex (female)	35 (74.5%)		34 (75.6%)		0.904
Age (years)		74.94 ± 6.03		74.76 ± 5.77	0.884
Height (cm)		156.17 ± 7.98		155.00 ± 7.51	0.471
Weight (kg)		62.97 ± 10.17		53.24 ± 7.69	<0.001
BMI (kg/m^2^)		25.75 ± 2.94		22.09 ± 2.12	<0.001
BMI range					<0.001
Underweight (BMI < 18.5 kg/m^2^)	0 (0%)		3 (6.7%)		
Healthy weight (18.5–23.9 kg/m^2^)	15 (31.9%)		33 (73.3%)		
Overweight (24.0–26.9 kg/m^2^)	15 (31.9%)		9 (20.0%)		
Obesity (BMI > 27.0 kg/m^2^)	17 (36.2%)		0 (0%)		
Physical performance					
Hand grip strength (kg)		21.49 ± 6.51		18.11 ± 6.63	0.016
Gait speed (m/s)		1.15 ± 0.46		1.13 ± 0.22	0.798
Body composition					
Body fat percentage (%)		37.66 ± 5.97		35.10 ± 6.73	0.056
Skeletal muscle index (kg/m^2^)		6.69 ± 0.78		5.61 ± 0.58	<0.001
Lean mass of upper limbs (kg)					
Left		2.182 ± 0.479		1.739 ± 0.429	<0.001
Right		2.198 ± 0.502		1.771 ± 0.394	<0.001
Lean mass of lower limbs (kg)					
Left		6.003 ± 1.141		5.038 ± 0.976	<0.001
Right		5.966 ± 1.087		4.985 ± 0.942	<0.001
Bone mass of upper limbs (kg)					
Left		0.119 ± 0.032		0.106 ± 0.037	0.075
Right		0.124 ± 0.041		0.114 ± 0.039	0.208
Bone mass of lower limbs (kg)					
Left		0.365 ± 0.095		0.324 ± 0.087	0.038
Right		0.366 ± 0.089		0.325 ± 0.088	0.029

To analyse whether any of the identified metabolites or lipids are associated with sarcopenia, we carried out a univariate and multivariate analysis of the identified metabolites and lipids. The OPLS‐DA score plots from the multivariate analysis of untargeted metabolomics in NMR and LC–MS are shown in Figure S1A (NMR) and 1B (LC–MS), and lipidomics in LC–MS (Figure S1C). The OPLS‐DA plots showed two clearly separated clusters, suggesting that the plasma metabolomes and lipidomes differed between the two groups.

### Identification of potential metabolites with discriminative features

**Figure 1:**
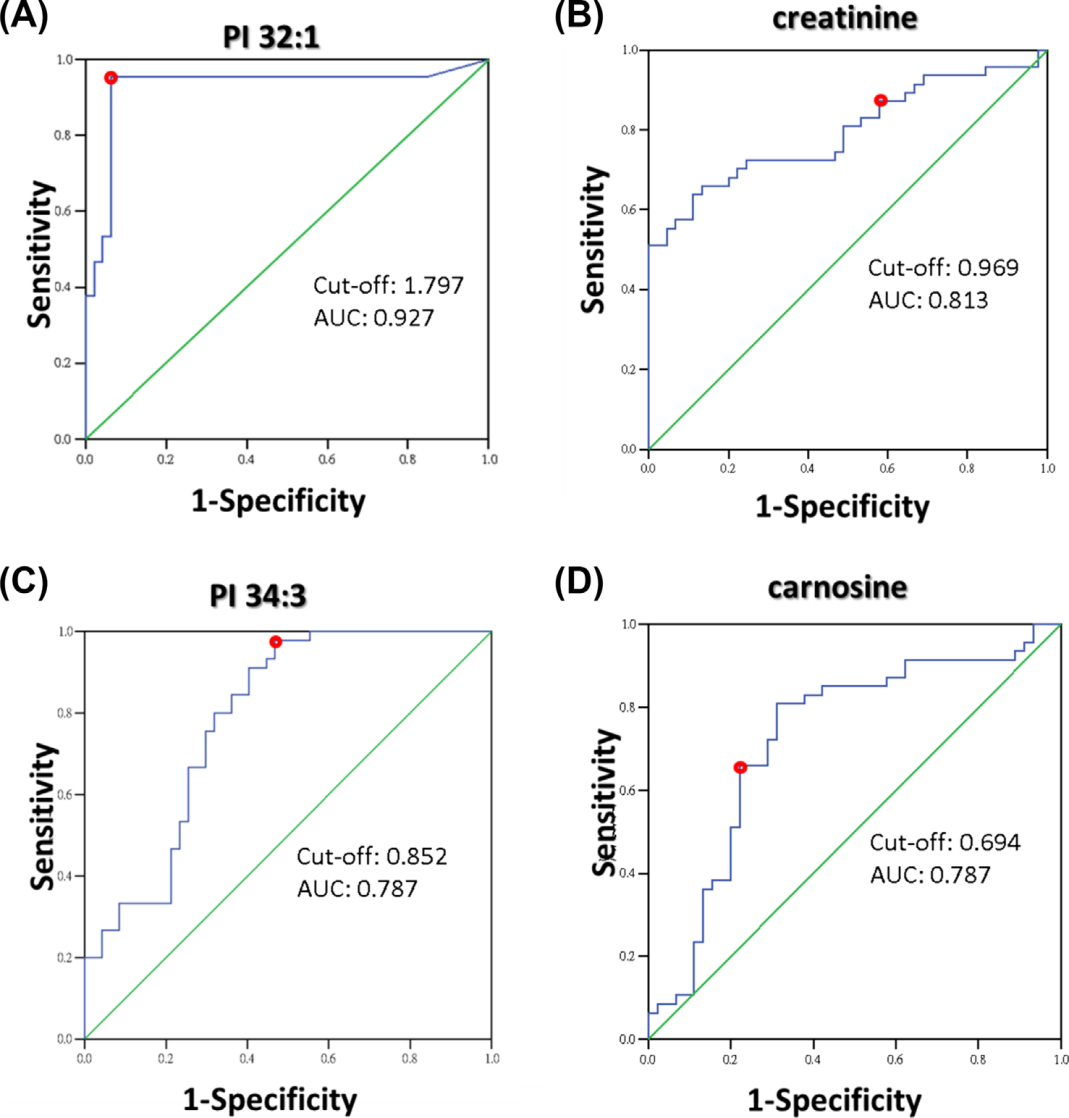
ROC curve of PI32:1, creatinine, PI34:3, and carnosine. The red dots denote cut‐off value. AUC, area under the receiver operating characteristic curve.

The Student's *t*‐test was firstly used to identify metabolites that differed significantly between patients and controls. Results showed that the untargeted metabolome and lipidome of sarcopenic and non‐sarcopenic plasma samples performed by ^1^H‐NMR and LC–MS identified 22 and 421 metabolites among the study individuals, of which 5 and 51 had statistically significant differences between two groups, but only 2 and 26 parameters were considered significant after ‘FDR’ adjustment (*P* < 0.05, FDR < 0.05), respectively (Table S1). Subsequently, VIP analysis was performed to determine the metabolites that made the greatest contribution, and ROC analysis was conducted to assess the metabolites with strong predictive power (AUC > 0.70). A criterion combining multiple data processing methods, including *P* < 0.05, FDR < 0.05, VIP > 1, and AUC > 0.70, was used to select potential biomarkers with good accuracy. Twelve plasma metabolites (isoleucine, carnitine, 1‐methylhistamine/3‐methylhistamine, creatinine, carnosine, ureidopropionic acid, uric acid, PC (18:2/20:0), PC (20:2/18:0), PC (18:1/20:1), PI 32:1, and PI 34:3) fulfilled these criteria (Table 2). In ROC curve analysis of the 12 metabolites, we obtained cut‐off values, sensitivities, specificities, and Youden indexes to diagnose sarcopenia. 1‐Methylhistamine/3‐methylhistamine, creatinine, ureidopropionic acid, PC (18:2/20:0), PI 32:1, and PI 34:3 had sensitivities larger than 0.75; carnosine, uric acid, and PI 32:1 had specificity larger than 0.75. Among them, PI 32:1, carnosine, creatinine, and PI 34:3 had Youden index larger than 0.5. PI 32:1 and carnosine had diagnostic accuracy for sarcopenia as high as 0.938 and 0.801, respectively, if we set 10% as an estimate for prevalence of sarcopenia (Figure 1). As to the relationship with skeletal muscle mass index (SMI), carnitine, 1‐methylhistamine/3‐methylhistamine, PI 32:1, and PI 34:3 correlated with SMI negatively; isoleucine, creatinine, carnosine, ureidopropionic acid, uric acid, PC (18:2/20:0), PC (20:2/18:0), and PC (18:1/20:1) correlated with SMI positively (Table 3).

**Table 2:** Potential metabolites involved in sarcopenia

Method	Features	HMDB ID	FC (patient/control)	*P* value (patient vs. control)	FDR value (patient vs. control)	VIP score (patient vs. control)	AUC (patient vs. control)	FC (post‐intervention/pre‐intervention)	*P* value (pre‐intervention vs. post‐intervention)
NMR	Isoleucine	HMDB0000172	0.86 ± 0.04	0.0005	0.011	1.063	0.7606	1.01 ± 0.70	0.9177
	Carnitine	HMDB0000062	1.05 ± 0.01	0.0110	0.060	1.070	0.7167	0.97 ± 0.05	0.0963
LC–MS	1‐Methylhistamine/3‐methylhistamine	HMDB0000898/HMDB0001861	1.24 ± 0.14	0.0039	0.004	1.054	0.7074	0.77 ± 0.03	0.0004
	Creatinine	HMDB0000562	0.71 ± 0.04	1.93E‐05	0.001	1.355	0.8127	1.089 ± 0.19	0.1092
	Carnosine	HMDB0000033	0.71 ± 0.04	0.0007	0.011	2.567	0.7867	1.32 ± 0.15	0.0423
	Ureidopropionic acid	HMDB0000026	0.61 ± 0.10	0.0107	0.034	3.309	0.7012	1.01 ± 0.67	0.9236
	Uric acid	HMDB0000289	0.88 ± 0.03	0.0083	0.037	1.512	0.7034	1.02 ± 0.24	0.6152
	PC (18:2/20:0)	HMDB0013436	0.69 ± 0.03	0.0010	0.046	2.369	0.7347	1.25 ± 0.07	0.0372
	PC (20:2/18:0)	HMDB0008333	0.70 ± 0.06	0.0014	0.047	2.333	0.7357	1.24 ± 0.32	0.0557
	PC (18:1/20:1)	HMDB0008077	0.74 ± 0.05	0.0015	0.042	2.065	0.7130	1.18 ± 0.13	0.1073
	PI 32:1	HMDB0009779	4.72 ± 0.17	0.0006	0.043	1.035	0.9272	0.28 ± 0.06	0.0020
	PI 34:3	HMDB0240614	1.88 ± 0.13	0.0003	0.039	1.006	0.7867	0.63 ± 0.11	0.0038

AUC, area under the receiver operating characteristic curve; FC, fold change; FDR, false discovery rate; PC, phosphatidylcholines; PI, phosphatidylinositol; VIP, variable importance in projection.

**Table 3:** Spearman's rank correlation coefficient with skeletal muscle mass index

	SMI	RUM	LUM	RLM	LLM
Isoleucine	0.342 (0.001)	0.052 (0.620)	0.084 (0.424)	0.095 (0.369)	0.068 (0.519)
Carnitine	−0.238 (0.022)	−0.313 (0.002)	−0.293 (0.005)	−0.298 (0.004)	−0.267 (0.010)
1‐Methylhistamine/3‐methylhistamine	−0.347 (0.001)	−0.015 (0.885)	−0.023 (0.826)	−0.050 (0.635)	−0.083 (0.403)
Creatinine	0.430 (0.000)	0.332 (0.001)	0.329 (0.001)	0.244 (0.019)	0.239 (0.022)
Carnosine	0.393 (0.005)	0.571 (0.000)	0.526 (0.000)	0.463 (0.000)	0.419 (0.002)
Ureidopropionic acid	0.219 (0.036)	0.070 (0.509)	0.071 (0.501)	0.068 (0.518)	−0.013 (0.906)
Uric acid	0.265 (0.011)	0.386 (0.000)	0.371 (0.000)	0.270 (0.009)	0.289 (0.005)
PC (18:2/20:0)	0.473 (0.000)	0.446 (0.001)	0.441 (0.001)	0.475 (0.000)	0.468 (0.000)
PC (20:2/18:0)	0.366 (0.000)	0.374 (0.000)	0.376 (0.000)	0.370 (0.000)	0.382 (0.000)
PC (18:1/20:1)	0.338 (0.001)	0.326 (0.002)	0.333 (0.001)	0.368 (0.000)	0.364 (0.000)
PI 32:1	−0.650 (0.000)	−0.391 (0.000)	−0.356 (0.000)	−0.421 (0.000)	−0.364 (0.000)
PI 34:3	−0.430 (0.000)	−0.340 (0.001)	−0.304 (0.003)	−0.316 (0.002)	−0.325 (0.002)

Numbers in parentheses denote *P* value.

LLM, muscle mass in left lower limb; LUM, muscle mass in left upper limb; RLM, muscle mass in right lower limb; RUM, muscle mass in right upper limb; SMI, skeletal muscle mass index.

**Figure 2:**
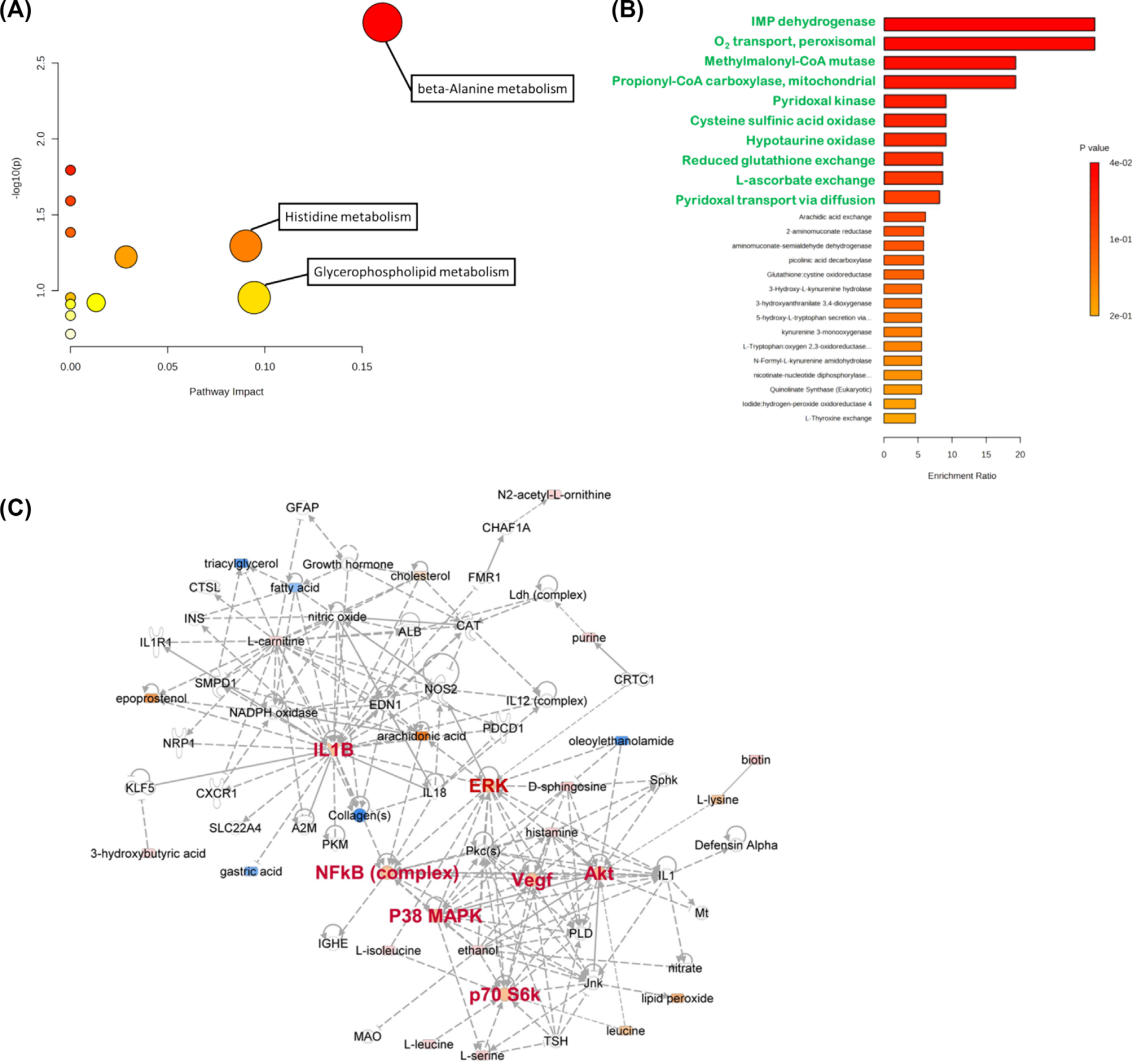
Summary of pathways related to sarcopenia and metabolite‐metabolite interaction network analysis. (A) Network pathway was identified by MetaboAnalyst. Metabolism was inferred from changes in levels of intermediates during substance metabolism in plasma. (B) Cellular enrichment analysis of interaction between changed metabolites and the enzymes. (C) Network pathways were identified by MetaboAnalyst software. Network analysis of differentially expressed metabolites was performed by ingenuity pathway tools (www.ingenuity.com) on metabolites annotated in the ingenuity database. Significant changes in amino acid metabolism, molecular transport, inflammatory response, free radical scavenging, and lipid metabolism, as well as PI3K‐AKT signalling, mTOR signalling, and ERK/MAPK signalling network were identified.

To reveal the most relevant pathways in sarcopenia, we used metabolic pathway analysis with MetaboAnalyst 5.0, which involves pathway analysis through pathway enrichment and topological pathway analyses. Pathway analysis revealed that three major metabolic pathways, beta‐alanine metabolism, histidine metabolism, and glycerophospholipid metabolism, may be disturbed in sarcopenia (impact > 0.1, *P* < 0.05) (Figure 2A). Based on metabolite set enrichment analysis, we found that these metabolites might affect functions of IMP dehydrogenase, O_2_ transport (peroxisomal), methylmalonyl‐CoA mutase, and propionyl‐CoA carboxylase (mitochondrial) (Figure 2B). Information on these metabolites was queried using the IPA software to establish metabolic networks. The results revealed that these differential metabolites were associated with amino acid metabolism, molecular transport, inflammatory responses, free radical scavenging, lipid metabolism, PI3K/AKT/mTOR and ERK/MAPK signalling (Figure 2C).

### Resistance training and nutritional support intervention improves sarcopenia

The primary treatment for sarcopenia is exercise, specifically resistance or strength training. These activities increase muscle strength and endurance through the use of resistance. The patient group exhibited a significant increase in grip strength (2.56 ± 5.19 kg) and skeletal muscle mass index (0.41 ± 0.45 kg/m^2^) after 12‐week lower‐limb RTNS. Both upper and lower limb lean mass increased significantly after bilateral exercise training (146 ± 369 g in the left upper limb, 156 ± 316 g in the right upper limb, 278 ± 325 g in the left lower limb, and 293 ± 376 g in the right lower limb) (Table 4). These data revealed that our RTNS programme effectively improved physical function and body composition in older adults with sarcopenia.

**Table 4:** Demographics, physical performance, and body composition before and after intervention (*n* = 45)

	Before	After	Difference (after‐before)	*P* value
	Mean ± SD	Mean ± SD	Mean ± SD	
Demographic				
Body mass index (kg/m^2^)	22.09 ± 2.12	21.83 ± 2.29	−0.27 ± 0.64	0.008
Physical performance				
Hand grip strength (kg)	18.11 ± 6.63	20.67 ± 5.84	2.56 ± 5.19	0.002
Gait speed (m/s)	1.13 ± 0.22	1.07 ± 0.20	−0.06 ± 0.24	0.084
Body composition				
Body fat percentage (%)	35.10 ± 6.73	34.62 ± 5.96	−0.48 ± 2.96	0.281
Skeletal muscle index (kg/m^2^)	5.61 ± 0.58	6.02 ± 0.76	0.41 ± 0.45	<0.001
Lean mass of upper limbs (g)				
Left	1739 ± 429	1885 ± 563	146 ± 369	0.011
Right	1771 ± 394	1928 ± 577	156 ± 316	0.002
Lean mass of lower limbs (g)				
Left	5038 ± 976	5317 ± 943	278 ± 325	<0.001
Right	4985 ± 942	5278 ± 926	293 ± 376	<0.001
Bone mass of upper limbs (g)				
Left	104 ± 35	110 ± 41	6 ± 15	0.002
Right	112 ± 39	118 ± 43	6 ± 15	0.004
Bone mass of lower limbs (g)				
Left	320 ± 83	327 ± 85	7 ± 17	0.001
Right	321 ± 84	330 ± 84	9 ± 18	<0.001

### Effects of resistance training and nutritional support for sarcopenia on untargeted metabolomic and lipidomic profiling performance

**Figure 3:**
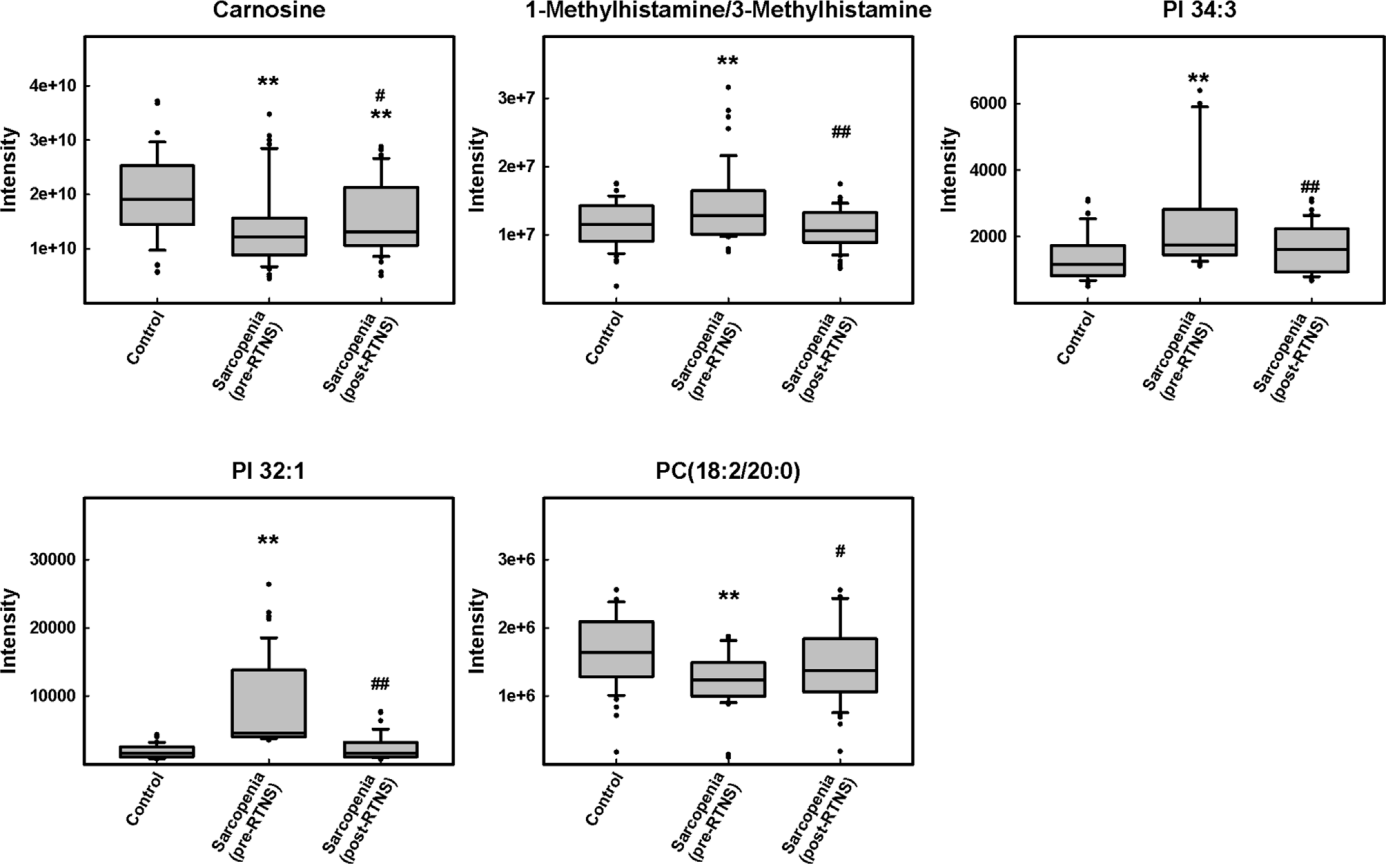
Levels of RTNS‐responsive metabolites in plasma of sarcopenic patients by LC‐QTOF‐MS and LC–MS/MS. Differential expression of RTNS‐responsive metabolites, 1‐methylhistamine/3‐methylhistamine, carnosine, PC (18:2/20:0), PI 32:1, and PI 34:3, in the plasma of patients with sarcopenia were shown. Data are mean ± SEM. *P < 0.05, **P < 0.01 compared with the control group. ^#^ *P* < 0.05, ^##^ *P* < 0.01 compared with the pre‐exercise group.

To obtain additional metabolic information, we compared pre‐ and post‐intervention metabolite levels in the sarcopenia group. Comparing pre‐ and post‐intervention levels, we found that many metabolites were markedly changed. A paired *t*‐test was used to select the metabolites with statistically significant changes (*P* < 0.05, pre‐ vs. post‐intervention in the sarcopenic group) (Table S2). We identified 31 metabolites with significant changes in patients with sarcopenia after intervention. Among the 31 identified metabolites, 10 metabolites with *P* values < 0.05 and FDR < 0.05 were considered statistically significant. It should be noted that five biosignatures, 1‐methylhistamine/3‐methylhistamine, carnosine, PC (18:2/20:0), PI 32:1, and PI 34:3, that were significantly altered in patients with sarcopenia could be significantly reversed after intervention (Figure 3).

## Discussion

Characterizing the metabolic profile associated with reduced muscle mass and strength in older adults holds significant translational implications for identifying subjects at a high risk of sarcopenia and developing early preventive strategies and treatments. Resistance training is an effective interventional strategy to attenuate and reverse the age‐related loss of muscle mass and strength.^18^ This study combined ^1^H‐NMR, LC‐QTOF‐MS, and LC–MS/MS to identify potential candidates for serum metabolomic and lipidomic signatures in older patients with sarcopenia. In addition, we observed metabolite changes that could be effectively reversed by RTNS in sarcopenia. To the best of our knowledge, this is the first study to explore biosignature changes in sarcopenia with RTNS using metabolomic and lipidomic approaches.

Recent metabolomic studies on sarcopenia indicated that some metabolites, such as L‐alanine, homocitrulline, and proline, were higher in the plasma of patients with sarcopenia, whereas plasma 4‐methyl‐2‐oxovaleric acid, 3‐methyl‐2‐oxovaleric acid, and tryptophan were lower in participants with severe sarcopenia than in non‐sarcopenic controls.^7^ Lower plasma concentrations of carnosine, branched‐chain amino acids (BCAAs), leucine, and isoleucine have been found in sarcopenic older individuals.^19^ Carnosine is abundantly present in the human skeletal muscle,^20^ and with age, the buffering capacity of muscles also declines due to reduced concentration of carnosine and sarcopenia.^21^ Additionally, some phosphatidylcholines (PCs), including PC diacyls, PC acyl‐alkyl, and lyso PC acyls, have been shown to be present at significantly lower concentrations in the plasma of participants with low muscle quality.^22^ In our study, we found that phosphatidylcholine 38:2 [including PC (18:2/20:0), PC (20:2/18:0), and PC (18:1/20:1)], carnosine, leucine, and isoleucine levels were significantly lower in participants with sarcopenia than in those without sarcopenia, and the levels of L‐alanine and proline were significantly higher in participants with sarcopenia than in those without sarcopenia (Table S1). The other four metabolites, carnitine, 1‐methylhistamine/3‐methylhistamine, PI 32:1, and PI 34:3, were significantly higher in the sarcopenia group than in the non‐sarcopenia group (Table 2).

BCAAs have been reported to participate in fatty acid metabolism and carnitine is essential for the transfer of long‐chain fatty acids across the inner mitochondrial membrane for subsequent β‐oxidation.^23^ In addition, some studies have shown that dietary BCAA supplementation is associated with an increase in PC biosynthesis,^24^ although the underlying mechanism is not clear. Moaddel et al.^22^ demonstrated that participants with low muscle quality have significantly lower PC concentrations. Our findings are consistent with his results (Tables 1, 2, and 3). Decreased PC levels in patients with lower muscle quality may result from dysfunctional mitochondria, which have been shown to play a role in muscle function decline.^25^ Interestingly, BCAAs are traditionally considered functional amino acids that regulate protein synthesis via the PI3K/AKT signalling pathway and mTOR complex1 (mTORC1), which controls the anabolic and catabolic signalling of skeletal muscle mass, resulting in the modulation of muscle hypertrophy and muscle wastage.^26^ Defects in PI3K/AKT/mTOR signalling have been observed in sarcopenic muscle,^27^, ^28^ and this could led to its substrate accumulation, including PI 32:1 and PI 34:3. Our results supported this hypothesis.^29^ Besides, muscle loss has been linked with several proteolytic systems, including the ubiquitin‐proteasome, lysosome‐autophagy, and TNF‐α/NF‐κB systems. These results are in accordance with the IPA results (Figure 2(C)).

Exercise and adequate nutrition are considered the main strategies for sarcopenia treatment. Resistance training can trigger a remodelling programme in skeletal muscles that progressively improves muscle mass and strength and enhances performance. It brings about gains in muscle size, strength, endurance, and power and is therefore recommended as the first‐line treatment for counteracting the deleterious consequences of sarcopenia in older adults.^30^ A single bout of resistance training can increase muscle protein synthesis within 2–3 hours and muscle protein synthesis remains high for up to 24 h in trained individuals,^31^ which can help improve muscle weakness. Gardner et al. showed that exercise training increases plasma carnosinase activity and decreases carnosine excretion, leading to increase muscle carnosine concentrations.^32^ Combining exercise with ingestion of dietary supplements, which are rich in carnosine, can slow the process of sarcopenia and aging.^21^, ^33^ Baumert et al. also found that carnosine levels decrease in skeletal muscle tissue during hypertrophy in mice, suggesting a higher demand for carnosine or its precursors, histidine, and/or β‐alanine.^34^ Furthermore, PC levels increased in the plasma of aged mice after exercise training.^35^ Both RT and aerobic training are beneficial for remedying sarcopenia by modulating the AKT/mTOR, AKT/FoxO3a, and AMPK signalling pathways,^22^, ^36^ as well as by enhancing BCAA uptake and absorption.^37^, ^38^ PI3K/AKT/mTOR signalling is a pivotal pathway that can induce skeletal muscle hypertrophy, and the activation of protein kinase B (PKB) can prevent muscle loss.^29^, ^30^ Although no evidence has shown that exercise could directly affect lipid metabolism, some studies have revealed that mTORC1 could stimulate PC synthesis to promote triglyceride secretion and regulate lipid homeostasis.^39^, ^40^ From the IPA analysis, we also found that the differential metabolites from our metabolomics and lipidomics analyses were associated with not only PI3K/AKT/mTOR and ERK/MAPK signalling but also amino acid metabolism, molecular transport, inflammatory responses, free radical scavenging, and lipid metabolism (Figure 2C).

This study has some limitations that should be considered when interpreting the findings. First, the study focused only on lower body resistance exercises and that the control group did not undergo the RTNS intervention. Second, the causality between the plasma metabolite levels and sarcopenia requires further investigation. Third, the relatively small sample size for other potential confounders, such as ethnicity, sex, clinical symptoms, complications (e.g., osteoporosis), or underlying diseases, might have limited the power of the study to identify sarcopenia trait‐related metabolites. Fourth, we could not exclude some unmeasured risk factors, such as eating habits, nutritional supplements, and lifestyle, even after adjusting for the available covariates. The lack of an assessment of dietary protein‐related parameters prevents a more comprehensive appraisal of this relationship. Fifth, we did not monitor participants' water intake, which might affect body composition measurement. Ideally, replication and larger‐scale studies are required to validate our findings. At last, since resistance training and nutritional support were provided simultaneously in this study, we cannot attribute the changes in metabolome and lipidome to exercise or nutrition only.

## Conclusion

This is the first research study to investigate changes in metabolomic and lipidomic profiles in sarcopenic patients following RTNS in older adults. Here, we identified 12 potential biomarkers of sarcopenia: isoleucine, carnitine, 1‐methylhistamine/3‐methylhistamine, creatinine, carnosine, ureidopropionic acid, uric acid, PC (18:2/20:0), PC (20:2/18:0), PC (18:1/20:1), PI 32:1, and PI 34:3. Among them, carnitine, creatinine, ureidopropionic acid, uric acid, PI 32:1, and PI 34:3 have not been previously described as biomarkers of sarcopenia. Additionally, we identified five potential biomarkers that could be reversed by our intervention. These five biomarkers, 1‐methylhistamine/3‐methylhistamine, carnosine, PC (18:2/20:0), PI 32:1, and PI 34:3, may play important roles in the exercise and nutrition treatment of sarcopenia. Our data provide new clues and valuable insights in this regard. Our findings provide novel insights and valuable clues in this context, warranting further validation in larger cohorts of sarcopenic patients.

## Funding

This work was supported by grants from National Science and Technology Council of Taiwan (108‐2321‐B‐001‐028‐MY2, 110‐2314‐B‐002‐060‐MY3, 111‐2321‐B‐001‐009, and 112‐2321‐B‐001‐009) and National Taiwan University Hospital Bei‐Hu Branch to D.S.H. (112 T002 and 11301), and NSTC 108‐2321‐B‐001‐028‐MY2 to Y.L.L.

## Conflict of interest

The authors have disclosed no potential conflicts of interest.

## Supporting information


**Figure S1.** OPLS‐DA analysis of metabolites in plasma from sarcopenic patients. 1H‐NMR‐ and LC–MS‐based metabolites were identified by metabolomic and lipidomic comparisons between controls and sarcopenic patients. OPLS‐DA plots are based on (A) 1H‐NMR data, (B) LC–MS data for the untargeted plasma metabolome, and (C) lipidome from control (light blue) and sarcopenic patients (red).

**Data S1.** Supporting Information.

**Table S1.** Supporting Information.

**Table S2.** Supporting Information.

## Data Availability

The data supporting the findings of this study are available from the corresponding authors upon request. We have shared all raw data in the website (https://figshare.com/s/95f1dccf2d96f0f31b29).
