# Supplementary figures and images for: Novel metabolic and lipidomic biomarkers of sarcopenia
Source: J Cachexia Sarcopenia Muscle. 2024 Aug 21;15(5):2175–86. doi: 10.1002/jcsm.13567 (PMC11446726; doi:10.1002/jcsm.13567)

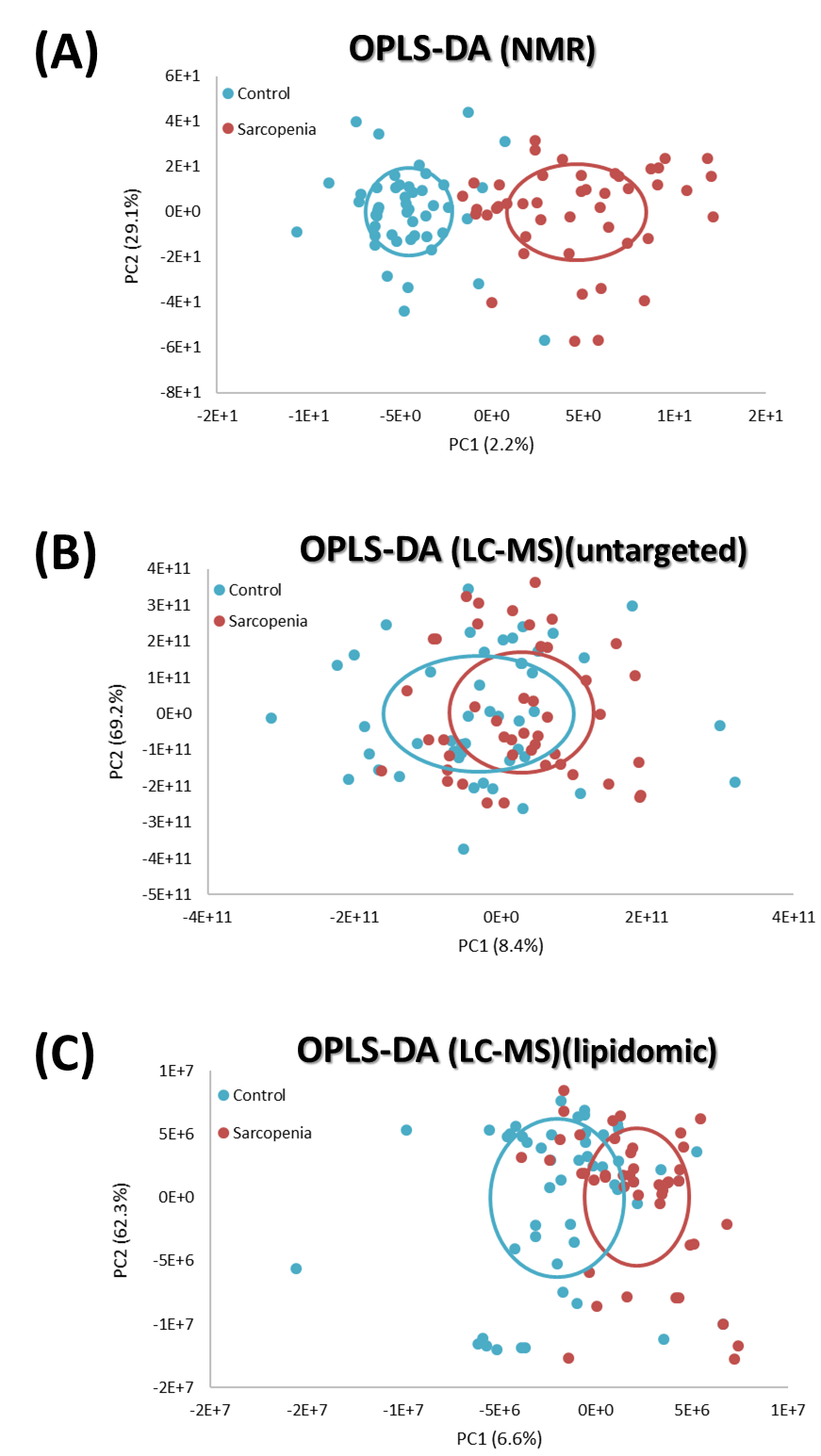

Supplement: Supplementary file 1 — Figure S1. OPLS‐DA analysis of metabolites in plasma from sarcopenic patients. 1H‐NMR‐ and LC–MS‐based metabolites were identified by metabolomic and lipidomic comparisons between controls and sarcopenic patients. OPLS‐DA plots are based on (A) 1H‐NMR data, (B) LC–MS data for the untargeted plasma metabolome, and (C) lipidome from control (light blue) and sarcopenic patients (red). [file JCSM-15-2175-s002.docx]
