# Supplementary material for: Novel metabolic and lipidomic biomarkers of sarcopenia
Source: J Cachexia Sarcopenia Muscle. 2024 Aug 21;15(5):2175–86. doi: 10.1002/jcsm.13567 (PMC11446726; doi:10.1002/jcsm.13567)
Supplement: Supplementary file 2 — Data S1. Supporting Information. [file JCSM-15-2175-s001.docx]

**Supplementary Materials and Methods**

**Grip strength**

Handgrip strength of dominant hand was measured using a handheld dynamometer from baseline. Participants were seated with their elbow flexed to 90 degrees during the examination and were instructed to grasp the dynamometer using their maximum strength three times with a one-minute interval between each test. The highest value obtained during the three attempts was recorded as the grip strength [1].

**Gait speed**

The study measured gait speed using the 5-meter walk test. Four markers were placed at specific points along a straight line measuring 7 m in length, including the starting point, 1 m, 6 m, and endpoint. Gait speed was calculated by dividing the distance of 5 m by the time taken to pass through the 1-meter and 6-meter markers. Participants were asked to walk comfortably during the test [1].

**Body composition**

The bone mineral content, fat mass, and lean body mass were measured using dual-energy X-ray absorptiometry (Stratos dR; DMS Group, France) [10]. The skeletal muscle mass index was calculated by dividing the lean muscle mass (kg) by the square of the height (m^2^). The machine projects a 2-dimensional fan-beam through a 256-pixel detector and takes approximately 30 s to scan one body part. The manufacturer's protocol was followed for the entire examination, and participants were asked to wear their gowns after undressing. Prior to the test, participants were instructed to fast overnight for at least 8 h. To estimate segmental body composition, the upper limb was separated from the trunk using a line connecting the axilla and acromioclavicular joint, whereas the inguinal skin fold was used as the border to distinguish the lower limb and trunk. The second cervical vertebra was considered the cranial level of the trunk; the head was not analyzed in this study [1].

**Reference:**

1. Chang KV, Wu WT, Huang KC, Han DS. Effectiveness of early versus delayed exercise and nutritional intervention on segmental body composition of sarcopenic elders - A randomized controlled trial. Clin Nutr. 2021;40:1052-9. doi:10.1016/j.clnu.2020.06.037
